# Familial benign recurrent acral pigmentation: A rare presentation

**DOI:** 10.1016/j.jdcr.2026.04.067

**Published:** 2026-05-12

**Authors:** Supriya Paudel, Himani Singh, Shreya Paudel

**Affiliations:** a Department of Dermatology, Madan Bhandari Academy of Health Sciences, Hetauda, Nepal; b Department of Dermatology, Shree Birendra Hospital, Kathmandu, Nepal; c Biomedical Engineer, College of Biomedical Engineering and Applied Sciences, Kathmandu, Nepal

**Keywords:** acral pigmentation, benign recurrent acral pigmentation, skin of color

## Introduction

Acral pigmentation may arise from a wide spectrum of etiologies, ranging from benign melanotic macules to serious conditions such as acral lentiginous melanoma.^1^ Benign recurrent acral pigmentation appears to be a rare, idiopathic, self-limited disorder characterized by recurrent, asymptomatic brown macules on the palms and soles that resolve spontaneously within weeks to months.^2^ To date, the reported cases have been sporadic. Herein, we describe what appears to be the familial occurrence of transient acral pigmentation, adding a new dimension to this uncommon presentation.

## Case report

A 36-year-old woman presented with multiple, asymptomatic, brownish macules over the plantar aspects of bilateral feet, noticed over the preceding 3 months. The lesions would appear spontaneously, persist for a few days to weeks, and then fade completely, occasionally recurring in new areas. There was no preceding erythema, trauma, or drug intake prior to eruption. Interestingly, her 2 children, 1-year-old daughter and 7-year-old son developed similar pigmentation over their soles during the same period. None had systemic symptoms, mucosal pigmentation, or nail changes. There was no relevant family history in previous generations.

**Fig 1:**
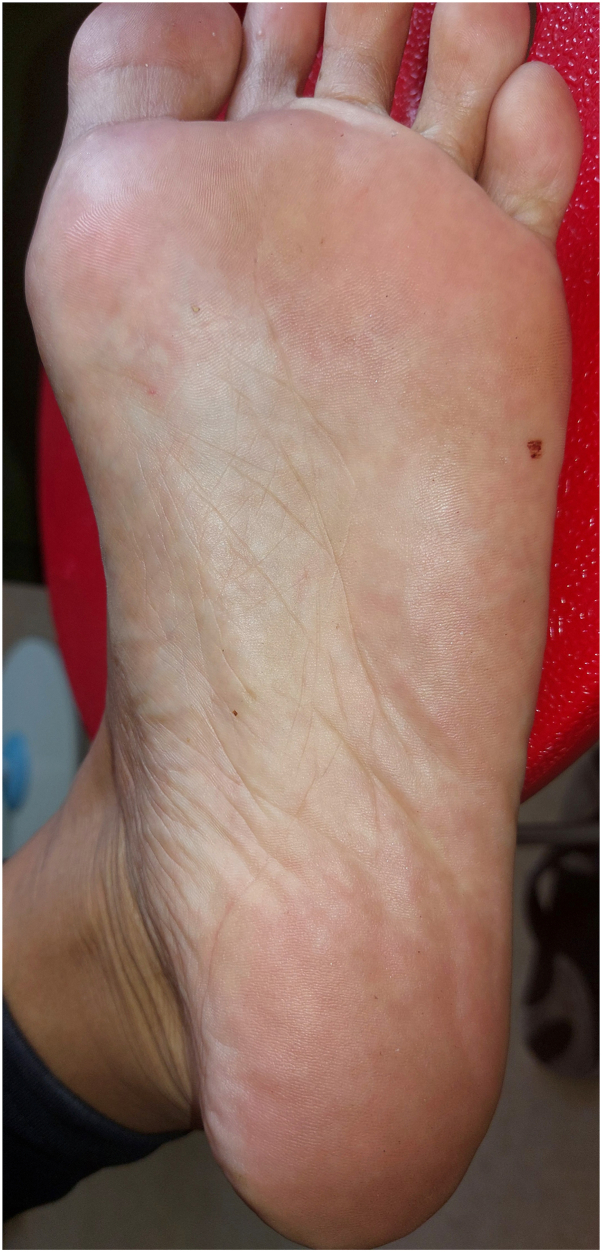
Solitary brown macule on the mother's left sole.

**Fig 2:**
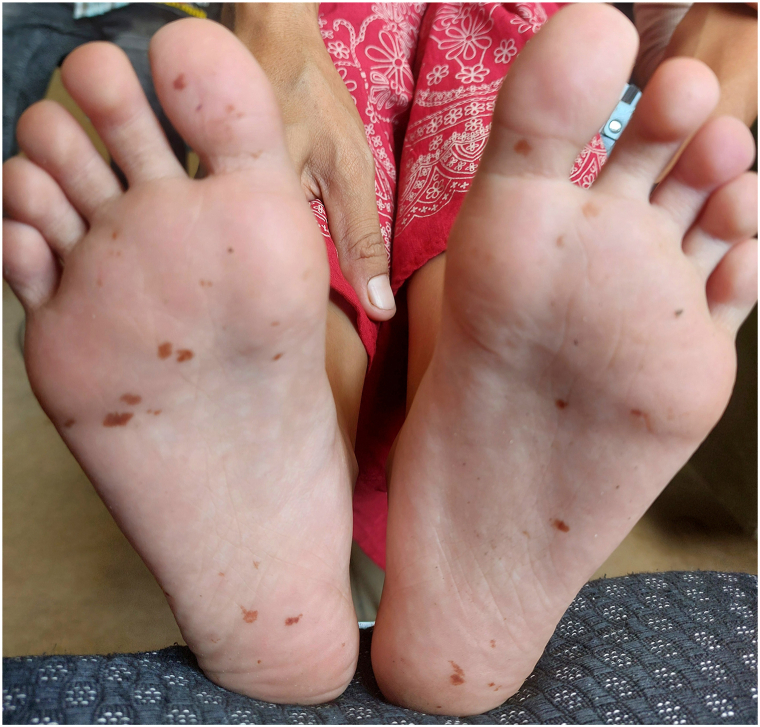
Multiple hyperpigmented macules on daughter's soles.

**Fig 3:**
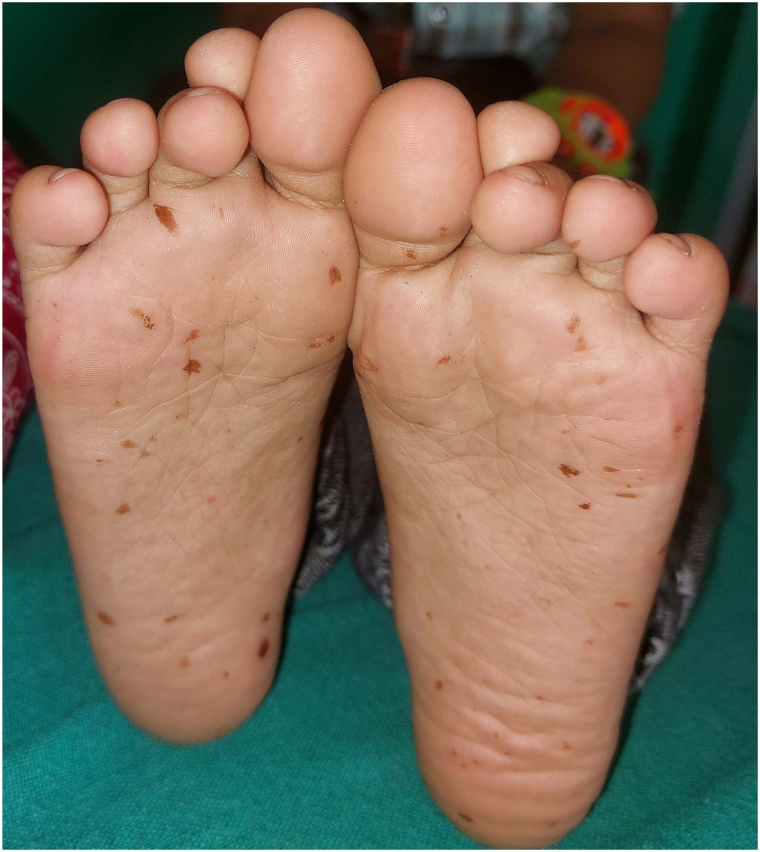
Multiple hyperpigmented macules on the son's soles.

**Fig 4:**
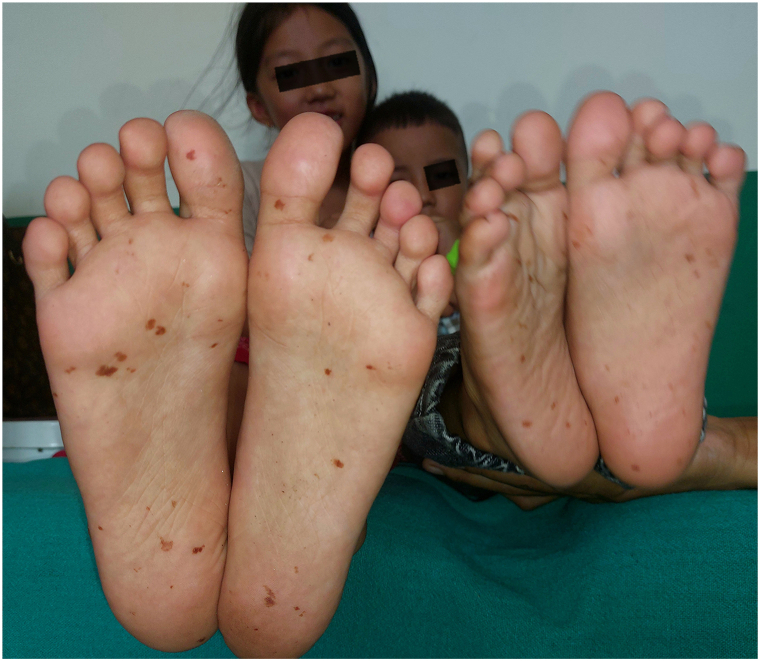
Multiple hyperpigmented macules on the soles of both the siblings.

On examination, all 3 had multiple, discrete, brown macules (2-5 mm) on the soles, with smooth surfaces, well-defined borders, and no scaling or induration (Fig 1, Fig 2, Fig 3, Fig 4). A wipe test with alcohol was negative, and KOH mount did not reveal fungal elements.

A clinical diagnosis of benign recurrent acral pigmentation was made. All were prescribed a topical moisturizer and advised follow-up; however, the family was subsequently lost to follow-up. No biopsy was performed as the patient denied.

## Discussion

Benign recurrent acral pigmentation is characterized by asymptomatic, transient, hyperpigmented macules over acral sites that resolve spontaneously without residual pigmentation. The etiology in this familial case remains unknown, though environmental, mechanical, and transient melanogenic triggers could have predisposed the lesions. The unique feature of the present report is the simultaneous occurrence in 3 members of a single family, suggesting either a shared environmental exposure (eg, transient exogenous pigment or irritant), a viral etiology or a genetic predisposition leading to heightened acral melanogenic response.^3^^,^^4^

The differential diagnoses considered were tinea nigra, exogenous pigmentation, and familial lentiginosis syndromes.^3^^,^^4^ However, they were ruled out by negative KOH, no indentifiable contact agent and absence of persistent mucosal involvement, respectively. Therefore, given the self-limited and recurrent course, benign recurrent acral pigmentation remains the most appropriate diagnosis, together with an atypical familial clustering. A limitation of this report is that dermoscopy was not performed on the lesions during the active phase, which might have aided in morphological characterization. No similar familial cases were found in literature to date.

This case highlights an unusual familial occurrence of benign recurrent acral pigmentation. It emphasizes the need to recognize transient acral pigmentation as a benign condition to avoid unnecessary investigations or interventions. Further reports and follow-up studies are warranted to determine whether familial clustering represents a genetic susceptibility or a shared environmental phenomenon.

## Conflicts of interest

None disclosed.
